# “Direct cloning in *Lactobacillus plantarum*: Electroporation with non-methylated plasmid DNA enhances transformation efficiency and makes shuttle vectors obsolete”

**DOI:** 10.1186/1475-2859-11-141

**Published:** 2012-10-25

**Authors:** Katharina Spath, Stefan Heinl, Reingard Grabherr

**Affiliations:** 1 Department of Biotechnology, Christian-Doppler-Laboratory for Genetically Engineered Lactic Acid Bacteria, Vienna Institute of Biotechnology, University of Natural Resources and Life Sciences, Muthgasse 11, Vienna 1190, Austria

**Keywords:** Lactobacillus plantarum, DNA methylation, mrr, Direct cloning, Library efficiency, Reduced plasmid size

## Abstract

**Background:**

Lactic acid bacteria (LAB) play an important role in agricultural as well as industrial biotechnology. Development of improved LAB strains using e.g. library approaches is often limited by low transformation efficiencies wherefore one reason could be differences in the DNA methylation patterns between the *Escherichia coli* intermediate host for plasmid amplification and the final LAB host. In the present study, we examined the influence of DNA methylation on transformation efficiency in LAB and developed a direct cloning approach for *Lactobacillus plantarum* CD033. Therefore, we propagated plasmid pCD256 in *E. coli* strains with different *dam*/*dcm*-methylation properties. The obtained plasmid DNA was purified and transformed into three different *L. plantarum* strains and a selection of other LAB species.

**Results:**

Best transformation efficiencies were obtained using the strain *L. plantarum* CD033 and non-methylated plasmid DNA. Thereby we achieved transformation efficiencies of ~ 10^9^ colony forming units/μg DNA in *L. plantarum* CD033 which is in the range of transformation efficiencies reached with *E. coli.* Based on these results, we directly transformed recombinant expression vectors received from PCR/ligation reactions into *L. plantarum* CD033, omitting plasmid amplification in *E. coli*. Also this approach was successful and yielded a sufficient number of recombinant clones.

**Conclusions:**

Transformation efficiency of *L. plantarum* CD033 was drastically increased when non-methylated plasmid DNA was used, providing the possibility to generate expression libraries in this organism. A direct cloning approach, whereby ligated PCR-products where successfully transformed directly into *L. plantarum* CD033, obviates the construction of shuttle vectors containing *E. coli*-specific sequences, as e.g. a ColEI origin of replication, and makes amplification of these vectors in *E. coli* obsolete. Thus, plasmid constructs become much smaller and occasional structural instability or mutagenesis during *E. coli* propagation is excluded. The results of our study provide new genetic tools for *L. plantarum* which will allow fast, forward and systems based genetic engineering of this species.

## Background

*Lactobacillus plantarum* and many other lactic acid bacteria (LAB) are “generally regarded as safe” (GRAS) organisms and possess the ability to efficiently secrete recombinant proteins directly into the culture medium. Thus, they are recognized as emerging candidates for the expression of recombinant proteins as well as for genetic and metabolic cell engineering, both in the fields of medical and industrial biotechnology [1,2]. *Lactococcus lactis* is to date the most widely used LAB strain for recombinant protein expression and was engineered to express cytokines [3], bacterial and viral antigens [4,5], membrane proteins [6] and enzymes [7]. *L. plantarum* plays an important role in many processes of animal feed industry [8]. For example, preservation of crop silage is often improved by adding starter cultures containing *L. plantarum* and other LAB. During the past decade the interest in developing new and genetically engineered LAB strains with improved properties has been continuously growing [9-13], e.g. to produce cellulose degrading enzyme activity [14]. *L. plantarum* has also been proved being a feasible expression host, recently expressing a ß-galactosidase from *Lactobacillus delbrueckii*[15] and a chitinase from *Bacillus licheniformis*[16].

Several vector systems exist [17-19], and different plasmid backbones and promoters are available for cloning and protein expression in LAB. Unlike for recombinant protein expression in *Escherichia coli*, the availability of different engineered LAB expression strains is limited. Often protein yields are very low, constraining the system to applications where small amounts are sufficient, e.g. to produce cellulose degrading enzyme activity [14]. In order to adapt a host’s metabolic capacities to the needs in biotechnology, the approach to use genetic engineering has been shown to successfully enhance product yield and quality in *E. coli*[20], *Saccharomyces cerevisiae*[21] and *Pichia pastoris*[22]. Also for *L. plantarum,* genetic engineering was shown to improve the metabolic performance, when mutations in sigma-factor (*rpoD*) conferred resistance against low pH conditions [13]. Often, these approaches are based on the generation of a great variety of genetically different clones and the subsequent screening for the desired phenotype. The strategy of cell engineering on a systems molecular level requires the possibility of high-throughput screening of a heterogenic pool of mutants. Thus, the generation of diverse genetic libraries is a prerequisite for fast and efficient host engineering. Also for protein engineering such as the adaptation of enzymatic activities to environmental conditions within a certain cell system, requires library based systems. While gene libraries can be generated in the size of 10^10^ in *E. coli*, and in the range of 10^7^ in *S. cerevisiae*, for LAB low transformation efficiencies are often a limiting factor. During the last decades many transformation protocols for LAB strains have been published. The successful introduction of plasmid DNA into LAB is dependent on strain specific features such as cell wall structure and composition plasmid size and the origin of replication.

In some LAB, the low number or even lack of transformants obtained after electroporation, may be attributed to various restriction modification (RM) systems encoded by the host. RM systems are widely spread in bacteria and serve the protection of invading DNA such as foreign plasmids or the DNA of bacteriophages. Most of these systems consist of a restriction enzyme and a corresponding methyltransferase that blocks the restriction activity, thus, protects the genome from self-cleavage (type I and III RM systems) [23]. In contrast, the type IV RM systems produce restriction enzymes which cleave solely methylated DNA. There are several reports, mostly referring to DNA adenine methylation (*dam*)/ DNA cytosine methylation (*dcm*) that methylation pattern of plasmid DNA has a major impact on transformation efficiency and allows plasmid DNA to circumvent host restriction mechanisms [24-26].

Since gene manipulation of expression vectors is much easier in *E. coli* than in LAB, and in order to gain sufficient amounts of plasmid DNA, normally, shuttle vectors are used to first build and propagate the final plasmid in *E. coli*. After subsequent purification the desired plasmid is then transformed into LAB. Many shuttle vectors are based on cryptic plasmids derived from LAB strains [27], which have been modified to contain both, the LAB specific and *E. coli* specific replicative elements. Often these shuttle vectors are structurally unstable either in *E. coli* or in the LAB-expression hosts, maybe due to their size or their chimeric nature, e.g. differences in GC-content (50% GC for *E. coli* versus 30 – 40% GC for LAB). Savijoki et al. [28] used *L. lactis* MG1363 as an intermediate host to circumvent such problems. However, this approach is limited to origins of replication functional in *L. lactis*. Another strategy is to design shuttle vectors that contain replicons which replicate in Gram (+) as well as in Gram (–) bacteria, e.g. based on the origin of replication of pWV01 or pSH71 [29,30]. Yet, due to their rolling circle-replication mechanism these plasmids tend to suffer from structural and segregational instability. Often, plasmids containing large DNA inserts cannot stably be maintained [31,32], and thus, rolling circle-replicating plasmids are only suitable for small genes. Therefore, cloning procedures would substantially improve by having plasmids available that are devoid of any *E. coli* derived sequences and are based on a stable origin of replication. We have previously compared several LAB strains in terms of transformation efficiency and plasmid stability. One of the tested *L. plantarum* (CD033) strains showed unexpectedly high transfection yields (6 x 10^5^ colony forming units (cfu)/μg DNA) [19]. Furthermore, this strain was found to be transformable with unmethylated DNA and therefore, became an interesting organism for further examinations regarding the influence of DNA methylation on transformation efficiency.

## Results and discussion

### Role of plasmid methylation in transformation of *L. plantarum* CD033

*L. plantarum* CD033 was tested for its ability to be transformed by plasmid pCD256 DNA prepared from four *E. coli* strains differing in their genotype regarding *dam*/*dcm*-methylation. The plasmid pCD256 is based on pUC19 containing the origin of replication of p256, originally isolated from *L. plantarum* NC7 [33], which was previously shown to be active in *L. plantarum* CD033 [19]. A gene encoding the chloramphenicol acetyl transferase (CAT) obtained from the *Staphylococcus aureus* plasmid pC194 served as a selection marker. *E. coli* strains JM109 (*dam*^+^, *dcm*^+^), BL21(DE3) (*dam*^+^, *dcm*^-^), GM33 (*dam*^-^, *dcm*^+^) and C2925 (*dam*^-^, *dcm*^-^) were used for plasmid propagation. The state of *dam*/*dcm*-methylation was confirmed by restriction analysis using *Dpn*II (blocked by *dam*-methylation) and *PspG*I (inhibited by *dcm*-methylation) (data not shown). After purification, we performed electrotransformation of *L. plantarum* CD033 and determined the number of cfu. Electroporation results are summarized in Table 1 and represent mean transformation efficiencies calculated from four independently performed transformations. Transformation efficiency for *dam*^+^/*dcm*^-^ pCD256 was slightly higher as compared to the transformation efficiency when pCD256 was *dam*^+^/*dcm*^+^ or *dam*^-^/*dcm*^+^. However, when pCD256 was propagated in the *dam*^-^/*dcm*^-^ strain *E. coli* C2925 transformation efficiency increased more than 1000-fold as compared to JM109 derived plasmids. The transformation efficiencies of nearly 10^9^ cfu/μg plasmids are comparable to commercially available *E. coli* cloning systems and succeed the efficiencies, normally achieved in yeast [34,35]. Thus, the possibility to produce libraries, may it be for systems based genetic engineering, evolutionary based enzyme engineering or randomization of promoter active sequences, has been made available in a LAB strain. Since many regulatory functions are conserved through-out different species of LAB, results from library screenings in *L. plantarum* CD033 might be transferred to other LAB strains and species. In addition to *L. plantarum* CD033, we further tested two other strains of the same species, *L. plantarum* CD032 and the type strain *L. plantarum* DSM20174. It turned out that the type strain could not be transformed using methylated plasmid at all, and with unmethylated plasmid the transformation efficiencies were lower as compared to the other two *L. plantarum* strains (Table 2). Also transformation efficiencies of *L. plantarum* CD032 were below the ones obtained with *L. plantarum* CD033. However, the tendency, to not only tolerate non-methylated DNA, but to give higher numbers of transformants/μg DNA than with methylated plasmids was confirmed. A reason for the tested *L. plantarum* strains prefering non-methylated DNA over methylated DNA could be that the latter is restricted by methyl-dependent restriction enzymes such as e.g. the Mrr type restriction proteins which belong to type IV restriction enzymes. We searched the whole genome data of *L. plantarum* strains available at the NCBI data base, *L. plantarum* WCFS1 which has just recently been resequenced and reannotated [36], the genome of *L. plantarum* JDM1 [37] and the complete genome of *L. plantarum* ST-III [38], for the presence of *mrr* (methylated adenine recognition and restriction) like genes. Based on the annotation of *L. plantarum* WCFS1 we found one *mrr* gene (GeneID:1061548). This gene (99% sequence identity) was also present in the genome sequences of *L. plantarum* JDM1 and of the type strain *L. plantarum* DSM20174 [39]; no putative *mrr* genes were detected in the genome of *L. plantarum* ST-III. BLAST analysis [40] of these genes revealed further identities (60% on amino acid level) to *Lactobacillus farciminis* KCTC 3681 and *Lactobacillus reuteri* 100-23, all other identities were below 60%. Sequence comparison revealed the presence of the conserved Mrr motifs within the N-terminal domain containing the catalytic activity [41]. Levels of homologies between lactic acid bacterial genes were found to be much higher than when comparison was performed including the *E. coli mrr* gene (25% identity), indicating that certain domains within the protein binding domain are conserved within the group of LAB. In *L. plantarum* CD033, the presence of an *mrr* gene was confirmed by PCR and subsequent sequencing of the obtained amplicon (data not shown). DNA sequence comparison revealed 99% identity of the *L. plantarum* CD033 gene with the *mrr* gene of *L. plantarum* DSM20174.

**Table 1:** **Transformation efficiency of *L. plantarum *CD033 with pCD256 isolated from different *E. coli *strains**

**DNA source**	**Relevant genotype**	**Transformation efficiency [cfu/μg DNA]**		
		**minimum**	**mean**	**maximum**
JM109	*dam+ dcm+*	2.5 x 10^5^	3.0 x 10^5^	5.0 x 10^5^
BL21 (DE3)	*dam+ dcm-*	3.0 x 10^5^	4.1 x 10^5^	4.7 x 10^5^
GM33	*dam- dcm+*	1.6 x 10^5^	2.3 x 10^5^	3.0 x 10^5^
C2925	*dam- dcm-*	5.0 x 10^8^	8.7 x 10^8^	9.7 x 10^8^

Transformation efficiencies are given as the minimum, the maximum and the arithmetic mean values of four replicates.

**Table 2:** Transformation efficiency of LAB strains transformed with variously methylated plasmid pCD256

**DNA source**	**Relevant genotype**	**Transfromation efficiency [cfu/μg DNA] of the following LAB strains**					
		***L. plantarum *CD032**	***L. plantarum *CD033**	***L. plantarum *DSM20174**	***L. buchneri *CD034**	***E. faecium *CD036**	*L. lactis *MG1363*
JM109	*dam*^+^*dcm*^+^	4.0 x 10^2^	3.0 x 10^5^	0	8.0 x 10^4^	20	2.0 x 10^5^
C2925	*dam*^-^*dcm*^-^	6.0 x 10^3^	8.7 x 10^8^	1.8 x 10^2^	0	0	0

Results are the arithmetic mean of four replicates. * transformed with pCDWV01.

Furthermore, three other LAB-species were tested for their susceptibility to be transformed by unmethylated plasmid DNA. *Lactobacillus buchneri* CD034 and *Enterococcus faecium* CD036 were shown previously to maintain the origin of replication from plasmid p256 [19], whereas for *L. lactis* MG1363 plasmid pCDWV01 [19], containing the origin of replication from the lactococcal plasmid pWV01, had to be used in order to provide replication, which has been shown to have similar stability properties as compared to pCD256 [19]. Results showed that for none of the performed electroporations colonies were received, possibly indicating restriction of non-methylated DNA (Table 2). RM systems have been described for different *L. lactis* strains [42] and appropriate genes have also been identified in the chromosome of *L. buchneri* CD034 [43], supporting this hypothesis. Furthermore, restriction analysis of plasmids obtained from *L. plantarum* CD033 indicated that no *dam*/*dcm*-methylation is present in this strain. Hence, we failed to introduce plasmid DNA deriving from *L. plantarum* CD033 into *L. buchneri* CD034 (data not shown). The suggestion arises, that insights in methylation patterns of other species might serve to in vitro-methylate ligation reactions or unmethylated plasmids in order to overcome the bottle neck of a specific host’s restriction system.

### Direct cloning in *L. plantarum* CD033

**Figure 1:**
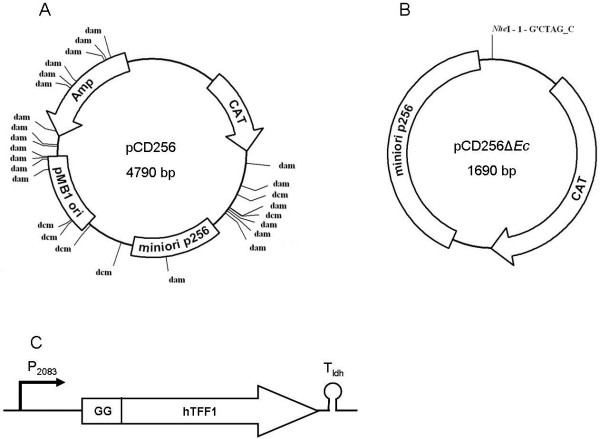
**Map of pCD256, pCD256Δ*Ec *and the synthetic *hTFF1 *expression cassette. A**: Map of pCD256 containing the minimal replicon (miniori) from plasmid p256 [33] for replication in LAB, pMB1 origin for replication in *E. coli*, a chloramphenicol resistance gene (CAT) for selection in LAB and an ampicillin resistance gene (Amp) for selection in *E. coli*. The *dam/dcm*-methylation sites are indicated. **B**: Map of the minimal plasmid pCD256Δ*Ec* consisting exclusively of the LAB-minimal origin from plasmid p256 and the chloramphenicol resistance gene for selection in LAB. **C**: Map of the synthesised *hTFF1* expression cassette consisting of the optimized *hTFF1*-gene, N-terminally fused to the *L. plantarum* CD032 *plnI* double glycine leader sequence (GG), promoter P_2083_ and terminator T_ldh_.

In order to see whether a transformation efficiency of up to almost 10^9^ cfu would be sufficient for direct cloning, a ligation reaction consisting of the vector backbone and a target gene was directly transformed into *L. plantarum* CD033 by electroporation. First, the vector pCD256 was propagated in *E. coli* C2925 (*dam*^-^/*dcm*^-^), purified and digested. The gene for the human trefoil factor 1 (*hTFF1*) served as the model target gene, as it is of small size (213 bps) and encodes a protein that being expressed in a food grade organism would be of high benefit. The codon optimized, synthetic *hTFF1* gene was amplified by PCR, cleaved and ligated into pCD256 to be expressed under control of the P_2083_ promoter. The ligation mix was directly transformed into *L. plantarum* CD033 by electroporation resulting in 10 colonies. PCR screening revealed that all clones contained the *hTFF1* gene. Thus, the step of propagating the LAB expression plasmid first in *E. coli* could be dismissed, making the requirement for an *E. coli/L. plantarum* shuttle vector obsolete. In order to proof this, the *E. coli* specific elements were deleted by PCR amplification maintaining only the *Lactobacillus* specific sequences of pCD256, resulting in a 1690 bp DNA fragment. Self-ligation of this DNA fragment resulted in plasmid pCD256Δ*Ec*, which now was 3100 bp smaller than pCD256 (Figure 1). Transformation into *L. plantarum* CD033 yielded 2,5 x 10^5^ cfu/250 ng vector DNA used in ligation mix. Given the fact that the intramolecular ligation reaction of linear fragments is very efficient, this high number of cfu is not surprising. Now, in order to test the feasibility of this approach also for direct cloning of a target gene, we linearized pCD256Δ*Ec* by PCR and after digestion performed ligation with the synthetic *hTFF1* expression cassette (Figure 1C) digested with the same enzymes. Direct transformation of the ligation reaction into *L. plantarum* CD033 yielded 5380 cfu/ligation reaction. This is a markedly higher number than when a vector backbone containing *E. coli* specific elements such as the origin of replication and the ß-lactamase gene was used. Identity of the plasmid and its structural integrity were confirmed by PCR amplification of overlapping plasmid fragments and subsequent DNA sequencing. So, it was shown that *L. plantarum* CD033 is the most suitable strain of the as yet tested LAB strains for direct cloning of recombinant expression vectors, circumventing the intricate design of shuttle vectors that are compatible with *E. coli*. Thus, plasmids become smaller, are devoid of unwanted sequences and occasional mutagenesis during *E. coli* propagation is excluded.

## Conclusions

In the present study, we examined the influence of DNA methylation patterns on transformation efficiency in LAB and developed a direct cloning approach for *L. plantarum* CD033. Therefore, we transformed various LAB strains with plasmid DNA exhibiting different *dam*/*dcm*-methylation patterns. Best results were obtained using non-methylated DNA, resulting in transformation efficiencies of ~ 10^9^ cfu/μg DNA in *L. plantarum* CD033. Thereby, it becomes feasible to generate expression libraries, promoter libraries, etc. of sufficient size in this strain. We demonstrated direct transformation of recombinant expression vectors received from PCR/ligation reactions into *L. plantarum* CD033 to be feasible, making the construction of shuttle vectors obsolete. This new approach allowed the construction of minimal plasmids consisting exclusively of a LAB-origin of replication and a selection marker. Besides providing smaller expression vectors, this method excludes any structural instability or mutagenesis which is occasionally associated with plasmid propagation in *E. coli*. The results of our study provide new genetic tools for *L. plantarum* which allow faster and facilitated cloning procedures as well as systems based genetic engineering based on library techniques.

## Materials and methods

### Bacterial strains and growth conditions

Bacterial strains used in this work are listed in Table 3. LAB were cultured in De Man-Rogosa-Sharp (MRS) medium [44] at 37°C for *L. buchneri* CD034, *L. lactis* MG1363 and *E. faecium* CD036 or at 30°C for *L. plantarum* strains. *E. coli* strains were grown in Luria-Bertani (LB) medium under continuous agitation at 37°C. For selection, the medium was supplemented with the appropriate antibiotics used at the following concentrations: ampicillin 100 μg/ml for *E. coli*, chloramphenicol 5 μg/ml for the LAB strains.

**Table 3:** Bacterial strains used in this study

**Strains**	**Endogenous methylation**		**Particular phenotypes**	**Source**
	***dam***	***dcm***		
**Escherichia coli**				
JM109	+	+		New England Biolabs GmbH
BL21 (DE3)	+	-		New England Biolabs GmbH
GM33	-	+	*dam*-3	M. G. Marinus
C2925	-	-	*dcm*-6/*dam*13::Tn9	New England Biolabs GmbH
**Lactic acid bacteria**				
*L. plantarum* CD032			wilde type strain	stable grass silage accession number BT6146*
*L. plantarum* CD033			wilde type strain	stable grass silage accession number BT6326*
*L. buchneri* CD034			wilde type strain	stable grass silage accession number BT6327*
*E. faecium* CD036			wilde type strain	stable grass silage accession number BT6329*
*L. lactis* MG1363			Plasmid free derivative of SH4109	[45]
*L. plantarum* DSM20174			type strain	[39]

* deposited in the strain collection of the Institute of Molecular Biotechnology, Graz, University of Technology, A-8010, Graz, Austria.

### Plasmids, primers and synthetic expression cassette

The plasmids used in this work are listed in Table 4. The gene *hTFF1* encoding the human trefoilfactor 1 was codon optimized for *L. plantarum* WCFS1 using JCat ( http://www.jcat.de). An expression cassette consisting of the optimized *hTFF1*-gene, N-terminally fused to the *L. plantarum* CD032 *plnI* double glycine leader sequence, promoter P_2083_ and terminator T_ldh_ was designed and synthesized by GeneArt (Life technologies, USA) (Figure 1 C)*.* P_2083_ was derived from *L.buchneri* CD034. It was found to drive transcription of the CAT gene in *L. plantarum* CD033 in a preliminary experiment and was later identified upstream of a gene encoding a putative fumarylacetoacetate hydrolase (LBUCD034_2083) in the *L. buchneri* CD034 genomic sequence [43]. Terminator T_ldh_ was derived from *Lactobacillus casei* BL23 (L-lactate dehydrogenase gene, LCABL-06930). Primers used in this study are listed in Table 5.

**Table 4:** Plasmids used in this study

**Plasmid**	**Size (bp)**	**Characteristics**	**Reference**
pCD256	4790	pUC19 containing origin from p256, Amp^R^, Cm^R^	[19]
pCDWV01	3514	pUC19 containing origin from pWV01, Amp^R^, Cm^R^	[19]
pCD256_*hTFF1*	5362	pCD256 containing *hTFF1* expression cassette	
pCD256Δ*Ec*	1690	pCD256 lacking *E. coli* sequences	this study
pCD256Δ*Ec*_*hTFF1*	2302	pCD256ΔEc containing *hTFF1* expression cassette	

**Table 5:** Primers used for cloning

**Primer**	**Sequence 5’→3’**	**Tm [°C]**
mrr_Lp_F	ATGAGTTATAAGCGTTGGAATG	60
mrr_Lp_R	TCAATCTTGTTCATAATAATATGC	60
hTff1_SacI_F	CGAGAGCTCGAATTCAGGTGTGATC	66
Tldh_amp_R	CTGCTGGTCGACAAAAAGATTAAAAAAGCC	64
M13_R_NheI	CGACGAGCTAGCAGCCAGGAAACAGCTATGACC	64
Cat_F_NheI	CGACGAGCTAGCAATGTGGTCTTTATTCTTCAAC	58
M13_R_XhoI	CGACGACTCGAGAGCCAGGAAACAGCTATGACC	64
Cat_F_NheI	CGACGAGCTAGCAATGTGGTCTTTATTCTTCAAC	58
hTff1_NheI_F	CGACGAGCTAGCGAATTCAGGTGTGATC	66
Tldh_amp_XhoI_R	CTGCTGCTCGAGAAAAAGATTAAAAAAGCCGCTGC	62
CAT_seq_R	AGTCATTCTTTACAGGAGTCC	60
GG_hTFF1_sense	ATGAAGATCAAGTTAACTGTTTTAA	62
CAT_seq_back	GTTATTGGGATAAGTTAGAGC	58
p256miniori_for	CCCGCACGCATAGCGGTGC	66

Restriction sites are underlined. Tm (melting temperature) was calculated with the program Gene Runner (Version 3.05, Hastings Software Inc., USA).

### PCR, restriction digestion and ligation of DNA fragments

Unless otherwise stated, DNA fragments were amplified using the Phusion High-Fidelity DNA Polymerase in HF-buffer (New England Biolabs, NEB, USA). PCRs were performed as follows: initial denaturation for 30 s at 98°C, followed by 30 cycles of 10 s at 98°C, annealing for 20 s at a melting temperature (Tm) +3°C of the lower Tm primer and extension for 25 s/kb at 72°C. Amplification was concluded with a final extension step at 72°C for 6 min. All PCRs were carried out with a T3 Thermocycler (Biometra, Germany). All restriction enzymes were purchased from NEB, restriction digests were performed according to the manufacturer’s recommendations. DNA fragments were purified from PCRs, enzyme reactions or agarose gels using the NucleoSpin Gel and PCR Clean-up Kit (Macherey-Nagel, Germany). Ligation reactions were performed using T4 DNA Ligase (NEB, USA). For a 20 μl ligation reaction 250 ng digested and purified plasmid DNA was mixed on ice with 1 μl T4 DNA ligase, 2 μl of 10x T4 ligase buffer and with a 5-fold molar excess of digested and purified insert DNA. The ligation reaction was incubated at 16°C over night precipitated with isopropanol, washed with 70% v/v ethanol, air dried for 15 min, solved in sterile ddH_2_0 and used for transformation.

### Dedection of the ***mrr*** gene in *L. plantarum* CD033

The presence of an *mrr* gene was confirmed by colony PCR using the Phusion High-Fidelity DNA Polymerase using GC-buffer (NEB, USA) and the primers mrr_Lp_F / mrr_Lp_R. PCR was conducted as follows: initial denaturation at 98°C for 30 s, followed by 30 cycles of denaturation at 98°C for 10 s, annealing at 60°C for 20 s and elongation at 72°C for 30 s. Cycling was completed with a final extension step at 72°C for 6 min. The amplicon was sequenced (GATC Biotech, Germany) and confirmed by BLAST analysis [40].

### Construction of pCD256_hTFF1, pCD256Δ*Ec* and pCD256Δ*Ec*_hTFF1

The synthetic *hTFF1* expression cassette was amplified by PCR using the primers hTFF1_SacI_F / Tldh_amp_R and cloned *Sac*I/*Sal*I into pCD256 resulting in the plasmid pCD256_hTFF1.

For the construction of pCD256Δ*Ec*, a DNA fragment comprising only lactobacillus specific elements (CAT, miniori256) was amplified from pCD256 by PCR using the primers M13_R_NheI / Cat_F_NheI, thereby introducing a novel *Nhe*I restriction site. The obtained fragment was digested with *Nhe*I and subsequently self-ligated.

For the ligation of two PCR products, pCD256Δ*Ec* and the *hTFF1* expression cassette were amplified by PCR using the primers M13_R_XhoI / Cat_F_NheI and hTFF1_F_NheI / Tldh_amp_R_XhoI. The two PCR products were *Xho*I/*Nhe*I-digested and ligated one with each other, resulting in the plasmid pCD256Δ*Ec*_*hTFF1*. DNA-cloning techniques in *E. coli* were performed according to Sambrook and Russell [46]. All vector constructs were confirmed by DNA sequencing (GATC Biotech, Germany).

### Electroporation of LAB

Electroporation was done using an ECM 630 Precision Pulse (BTX Harvard apparatus, USA). *L. plantarum* CD033 and *L. buchneri* CD034 were transformed according to [19], *L. plantarum* DSM20174 was transformed using the same protocol as for *L. plantarum* CD033, *L. lactis* MG1363 was transformed according to [47], *E. faecium* CD036 and *L. plantarum* CD032 were transformed as follows. An overnight culture was diluted with MRS broth to an optical density at 600 nm wavelength (OD_600_) of 0.2 and incubated at the appropriate temperature until an OD_600_ of 0.5 was reached. The cells were harvested by centrifugation at 10,000 rcf for 6 min at 4°C. The pellets were washed three times with ice cold 0.3 M sucrose. The pellet was resuspended to one hundredth of initial volume in 0.3 M sucrose and kept on ice until electroporation. Plasmid DNA (0.25-1 μg) was mixed with 40 μl cell suspension in a ice-cold electroporation cuvette (0.2 cm gap, Sigma Aldrich, Germany) and electroporated at 2.5 kV, 200 Ω and 25 μF. After the pulse, cells were resupended in 360 μl MRS broth for 2 h at the appropriate temperature. The bacteria were spread on MRS plates containing chloramphenicol and incubated for 3 days at the appropriate conditions. Proper assemble of recombinant plasmids was confirmed by colony PCR and supsequent sequencing of the obtained amplicons using the primers CAT_seq_back/p256miniori_for. In the case of pCD256Δ*Ec*_hTFF1 which was transformed directly with a purified ligation reaction, structural integrity was proven by PCR using the primers CAT_seq_R/Tldh_amp_R and CAT_seq_back/GG_hTFF1_sense yielding overlapping DNA fragments and subsequent DNA sequencing of the obtained fragments.

## Competing interests

The authors declare that they have no competing interests.

## Authors’ contributions

The work presented here was carried out in collaboration between all authors. KS, SH and RG defined the research theme and designed the experiments. KS carried out the laboratory experiments, analyzed the data, interpreted the results and prepared this manuscript with input, feedback and advice from SH and RG*.* All authors have contributed to, seen and approved the manuscript.
